# Phlegmasia Dolens Relieved by Cold Water

**Published:** 1853-09

**Authors:** 


*Phlegmasia Dolens Relieved by Cold Water.* The following case, isolated as presented, may not be of very great interest or importance, either to yourself or the readers of your most excellent journal; for which reason I would not even claim for it a place among the many instructive productions found on its pages. If I succeed by the report of this case in elicitating from you or your contributors an opinion in regard to the treatment adopted, my object will be fully accomplished.

And as the object of an editor is to collect from every possible source, materials out of which to form conclusions and to arrive at *facts*; which, when expressed to the profession, we feel under obligations to receive as such, founded upon true data, perhaps this may be of some service in making your collection of cases.

On the 2nd of April, 1852, I was called to attend Mrs. A. in her first confinement. The labor progressed rapidly, and she was soon delivered of a fine, healthy child, with no untoward symptoms except flooding, which, after great prostration, was arrested by the ordinary remedies. She was doing well up to the 9th, when she complained of a pain, which she described as a "cramping pain," in the calf of the right leg; this continued to increase during the following twenty-four hours, until it became of the most excruciating character; then successively the thigh, groin, and hip became affected, the pain becoming more severe as the disease advanced; at the same time the limb was hot and swollen; in short, I might say that there were present all the symptoms of a veritable case of *phlegmasia dolens*, perhaps more properly termed crural phlebitis, commencing as it sometimes, but not frequently, does at the lower instead of the upper part of the limb. This case was treated in the ordinary way with the exception that general depletion was not resorted to, which was inadmissable, on account of the great debility occasioned by excessive flooding at the time of her accouchment. The remedies seemed merely to act as palliatives, without checking the progress of the disease; for on the 20th the same symptoms began to make their appearance in the left leg that had been complained of in the right.

Being satisfied that if my patient was to suffer again what she had just passed through, she must certainly succumb, (for it had already become necessary to use stimulants pretty freely,) I determined upon a different course of treatment. I ordered a tub of the coldest spring water, directing it should be constantly poured upon the left leg for half an hour, after which wet cloths were to be applied for the same length of time. These applications were made to the whole limb, for the thigh had now become affected.

The next day my patient informed me that the limb to which the water had been applied felt much better, though it was still very painful, and I discovered, on examination, that the redness along the course of the vessels and swelling had somewhat subsided. The right leg was still painful. I directed the same application to both limbs to be repeated at least twice during the day; which was followed by very great relief. Indeed, it was only repeated for four successive days, when the inflammatory action had entirely subsided, and my patient was free from pain. It is unnecessary to state that her recovery was speedy from this date.

Without comment, I leave it with you and the profession to decide upon the propriety of the indiscriminate use of cold water in such cases, before the cessation of the lodhial discharge.

In this case there were no bad effects, no suppression of the discharge, but what the consequence of its application at the onset of the disease might have been I do not pretend to say. I also leave the case for the blind exultation of the Hydropathist, without going into an argument to prove that the use of water in this case is not empirical, but that it is scientific practice, founded upon the true pathology of the disease, which is essentially of an inflammatory character, whether this inflammation be seated in the absorbent or venous system.—*New Orleans Medical and Surgical Journal.*

